# Twenty-fifth Annual Meeting of the Ohio Slate Dental Society

**Published:** 1892-01

**Authors:** Otto Arnold


### PROCEEDINGS.

### Minutes of 25th Annual Meeting of the Ohio State Dental Society, at Hotel Chittendon, Columbus, Ohio, Wednesday, December 1st., 1891.

President E. G. Betty called the socicty to order at 10:30 A. M.

Minutes of last session read and approved, and roll of members called.

Chairman of the Executive Committee made an oral report of the arrangements perfected for this meeting. Report adopted.

None of the Membership Committee being present at this hour the president appointed Drs. Harroun, Butler and Lupton to act in that capacity.

Dr. Emminger stated, that in his opinion, the time had arrived when this body should cease doing missionary work, and therefore he moved that all other than members in good standing and invited guests should be excluded from the clinics and other privileges of this society.

Motion seconded by Dr. Harroun.

Dr. Sillito offered an amendment, seconded by Dr. Butler, to have the matter referred to the Board of Directors. Amendment carried.

Dr. Butler moved the time for sessions be from 9 A. M. to 11:30 A. M., 2 P. M. to 5 P. M., except Wednesday afternoon—the whole of which should be devoted to clinics. Carried.

Prsident Betty then read his address after which some miscellaneous matters were disposed of.

Two members of the Publication and Voluntary Essay Committee, being as yet absent, the president appointed Drs. J. Taft and O. M. Heise to complete the same.

There being no further business at this hour—adjourned until 2 P. M.

Tuesday afternoon session called to order by President Betty at 2:30.

Minutes of previous session read and approved.

A recess was granted members for the payment of dues.

Minutes of the proceedings of the Board of Directors read.

The following persons were recommended for membership by the Board of Directors and, duly elected:

H. C. Brown, D.D.S., Gallipolis; Edwin Waddell, D.D.S., Greenfield; Horace A. Hubbard, D.D.S., Dayton; M. H. Evans, D.D.S., Franklin.

Dr. C. M. Wright, of Cincinnati, read a paper "Soliloquy of a Plastidule," the chief feature of which was a biological treatise on man.

There being two other papers on the programme which were expected to bear on many of the points contained in the paper, discussion on the latter was deferred until Tuesday morning, at which time the aforesaid papers would be presented.

Dr. Raffensperger, of Marion, then read a paper on The Care of the Teeth During Pregnancy and Lactation.

This paper was discussed by Drs. H. A. Smith, J. Taft, A. O. Rawls, C. M. Wright, C. R. Butler and C. H. Harroun.

Dr. W. S. Elliott, of N. Y., read a paper on "Erosion of the Teeth," the subject being afterward discussed by Dr. H. A. Smith and others.

Dr. G. W. Melotte, of Ithica N. Y., was accorded the privilege of the floor and proceeded to explain the so-called new method set forth in a circular sent out to the profession by Dr. F. M. Harris.

Briefly stated—it is a process of adding gold to gold without solder or high heat. The gold is rendered plastic by the addition of mercury, squeezing out the excess of mercury, as in the preparation of amalgam fillings. A portion of the plastic mass is placed upon the gold and the mercury evaporated by slow heat.

Dr. F. Y. Clark, of Saratoga Springs, N. Y., had used the method for banding gold plates fifty years ago, and it is known as the Old Fire Gilding Process.

Dr. W. S. Elliott also gave the society some useful and practical hints about constructing tooth crowns.

Adjourned to 9:30 Wednesday.

WEDNESDAY DEC. 2, 9:30 A. M.

Session call to order at 9:30 A. M. by President Betty.

Minutes of previous session read and approved.

Roll ordered called, and a recess granted for the payment of dues.

Dr. J. Taft moved that the roll of membership be revised. Carried.

The president appointed the secretary and treasurer a committee for that purpose.

The following applicants for membership having been recommended by the Board of Directors they were balloted for and elected: H. M. Kirk, D.D.S., J. H. Price, D.D.S., A. B. Fletcher, D.D.S., C. L. Smith, D.D.S., Columbus; J. H. Boger, D.D.S., Findlay; H. F. Harvey, D.D.S., W. T. Jackman, D.D.S., Cleveland; C. P. Gray, D.D.S., Madisonville.

A paper was read by Dr. S. D. Stewart, of Akron, on "The Philosophy of Nutrition with Legal Aspects."

Dr. A. O. Rawls, of Kentucky, followed with a paper on "Heredity."

These papers, including Dr. C. M. Wright's, were discussed by Dr. Harroun and others.

Dr. Melotte, of N. Y., presented the subject of Crown and Bridge Work, or Gold Restorations, in a graphic manner with illustrations and diagrams upon the blackboard.

Adjourned.

WEDNESDAY 8:30 P. M.

Society called to order by 2d Vice-President Geo. H. Wilson.

Reading of the minutes was dispensed with, and the following were elected to membership upon the recommendation of the Board of Directors:

D. Haight, Coshocton; H. E. Dunn, D.D.S., Warren; J. S. McCampbell, D.D.S., Xenia; B. F. Johnson, Camden; A. J. Bosant, D.D.S., Springfield; A. Jones, D.D.S., Lima; J. H. Weibel, D.D.S., J. F. Daugherty, D.D.S., Canton; J. C. Oldham, D.D.S., Springfield; L. P. Holbrook, D.D.S., Mt. Vernon; L. W. Ballard, Alliance.

Dr. H. H. Harrison of W. Va., read a paper on "Relationship."

Dr. J. Taft moved that a voluntary paper from Dr. W. H. Sedgwick be read at the banquet this evening. Carried.

A paper read by Dr. H. A. Smith on "Antiseptics for Sterilizing Dental Caries" brought forth an animated discussion.

Drs. Callahan, Arnold, H. T. Smith, H. A. Smith, Gray, J. Taft, Wright and Rawls taking part therein.

Adjourned.

THURSDAY DEC. 3RD 10:30 A. M.

Session called to order by President Betty.

Minutes of the previous session read and approved and the programme continued.

Dr. J. G. Junkerman, of Cincinnati, read an interesting paper on "What Causes Variety and Modification in the Character of Dental Caries."

Remarks followed by Drs. Wilson, Wright and H. A. Smith, being limited in the main to the endorsement of the essayists views.

Dr. Callahan, of Cincinnati, read a paper on "Combination Fillings." He (the essayist) taking the ground that in our State and local societies the matter presented should be largely of a practical character. That there was a tendency to neglect this valuable field and introduce the extremely scientific instead.

Drs. Wright and H. A. Smith followed with remarks, the former citing the evolution of the griddle makers as an argument against the exclusively practical proposition, Dr. Smith presenting teeth containing combination fillings.

Dr. J. Taft read a paper on "Dental Hygiene" which was disdussed by Drs. Dennis, Whitslar and Taft.

On motion the society proceeded to elect officers for the ensuing year with the following results:

President, C. R. Callahan, Cincinnati; 1st. Vice-President, Geo. H. Wilson, Painesville; 2nd Vice-President, Chas. Welch, Wilmington; Secretary, Otto Arnold, Columbus; Assistant Secretary, Henry Barnes, Cleveland; Treasurer, C. I. Keeley, Hamilton.

BOARD OF DIRECTORS FOR THREE YEARS.

C. H. Harroun, C. C. Miles, J. A. Lupton, W. H. Hague.

TWO YEARS.

W. D. Tremper, W. H. Whitslar.

BOARD OF DENTAL EXAMINERS.

C. R. Butler, E. G. Betty.

The president-elect was conducted to the chair and introduced to the society by Dr. C. M. Wright.

Dr. Butler, of Cleveland, then read a paper on "Septicemia," which was followed by the reading of Dr. Sedgwick's paper on "Dental Legislation," by the secretary. The latter paper was discussed by Drs. J. Taft, Harroun and Arnold.

The work being completed the society adjourned to meet in Columbus, the first Tuesday in December, 1892, to continue in session four days.

Altogether considered this was perhaps the most successful and profitable meeting, to those in attendance, in the history of this society. The attendance was large, the programme was varied, the interest was continuous. A general sentiment of good will and satisfaction seemed to characterize the whole assembly from beginning to end, and when the guests departed it was with the hearty assurance that they would come again next year.

OTTO ARNOLD, Secretary.

